# Recent advances in the management of osteoporosis

**DOI:** 10.12688/f1000research.10682.1

**Published:** 2017-05-05

**Authors:** Seiji Fukumoto, Toshio Matsumoto

**Affiliations:** 1 Department of Molecular Endocrinology, Fujii Memorial Institute of Medical Sciences, Tokushima University, 3-18-15 Kuramotocho, Tokushima, Tokushima, 770-8503, Japan

**Keywords:** osteoporosis, bisphosphonates, denosumab, teriparatide, abaloparatide, romosozumab

## Abstract

There has been substantial progress in the management of patients with osteoporosis and the prevention of osteoporotic fractures. Currently available strong anti-resorptive agents are bisphosphonates and an anti-receptor activator of nuclear factor-kappa B ligand (RANKL) antibody, denosumab. Although bisphosphonates and denosumab both inhibit bone resorption and prevent vertebral and non-vertebral fractures, their mechanisms of action are different. Whereas bisphosphonates’ effects on bone mineral density and fracture peak around 3 to 5 years and become plateaued, those of denosumab are maintained for up to 10 years. There are differences in the modes of action of these two drugs. Bisphosphonates accumulate on the mineralized bone surface and are released by the acid environment under osteoclastic bone resorption, whereas denosumab is not accumulated on bone but directly binds RANKL and inhibits its binding to the receptor RANK. Thus, the reduction in denosumab concentration 4 to 6 months after injection may enable RANK to bind to RANKL, where it is highly expressed, such as in damaged bone regions. As anabolic agents, only teriparatide has been available for a long time, but abaloparatide, a synthetic analog of PTHrP(1–34), is currently under development. Because of the difference in the preferential binding conformations of PTH1 receptor between teriparatide and abaloparatide, the latter shows anabolic effects with fewer bone resorptive effects. Romosozumab, an anti-sclerostin antibody, inhibits the action of sclerostin, a canonical Wnt signal inhibitor secreted from osteocytes, and enhances canonical Wnt signaling. Romosozumab robustly increases vertebral and proximal femoral bone mineral density within 12 months and inhibits vertebral and clinical fractures in patients with osteoporosis by enhancing bone formation and inhibiting bone resorption. In this review, we summarize the recent advances in therapeutic agents for the treatment of osteoporosis and discuss future prospects with their use.

## Introduction

Osteoporosis is a disease characterized by reduced bone mass and strength. Adult bone is continuously remodeled by osteoclastic bone resorption and osteoblastic bone formation. Advances in the treatment of osteoporosis have made substantial improvement in the management of patients with osteoporosis and the prevention of osteoporotic fractures. Many of these drugs increase bone mass and strength by inhibiting bone resorption, but there are drugs that increase bone formation. In this review, we summarize recent advances in the development of anti-osteoporotic drugs by focusing on bisphosphonates, denosumab, teriparatide, abaloparatide, and romosozumab. There are other drugs being used for the treatment of osteoporosis in some areas of the world but not discussed in this review, such as selective estrogen receptor modulators, active vitamin D compounds, vitamin K compound, strontium ranelate, calcitonin, and parathyroid hormone (PTH) (1–84).

## Bisphosphonates

The first bisphosphonate developed for the treatment of osteoporosis was etidronate, which had weak anti-resorptive activity compared with its mineralization inhibitory activity. Thereafter, nitrogen-containing bisphosphonates with much stronger anti-resorptive activity have become the main drugs for patients with osteoporosis. Bisphosphonates bind to hydroxyapatite during mineralization or demineralization and are released under acidic condition during osteoclastic bone resorption. Nitrogen-containing bisphosphonates suppress bone resorption by inhibiting the activities of osteoclasts and inducing their apoptosis. These drugs mainly increase the bone mineral density (BMD) of trabecular bones. For example, it has been shown that alendronate increased lumbar BMD for up to 10 years ^1^. In contrast, the increase of BMD in proximal femur or total body reached a plateau after 3 to 5 years ^1^. Similarly, the increase of proximal femoral BMD by once-yearly intravenous administration of zoledronic acid was evident for up to 4 years ^2^. However, femoral BMD remained stable after that period, even with continuing administration of zoledronic acid. In addition, phase 3 clinical trials have shown that several nitrogen-containing bisphosphonates prevent vertebral, non-vertebral, and proximal femoral fractures during treatment for 3 years. However, the effects of long-term administration of nitrogen-containing bisphosphonates on fracture prevention, especially of non-vertebral bones, are not clear. In a study comparing the effects of continuous administration of alendronate for 10 years and alendronate for 5 years followed by placebo for 5 years, clinical vertebral fractures were reduced in patients who received alendronate for 10 years ^3^. However, there was no difference in the frequency of morphometric vertebral fractures, non-vertebral fractures, proximal femoral fractures, and wrist fractures between patients who received alendronate for 10 years and 5 years ^3^. Similarly, there was no difference in the frequency of morphometric vertebral fractures and clinical fractures in patients who received intravenous zoledronic acid for 9 years and 6 years followed by placebo for 3 years ^2^, although fracture risk remained lower in the patients receiving zoledronic acid compared with those without zoledronic acid treatment. These results suggest that the efficacy of bisphosphonate on fracture prevention reaches a plateau after several years.

In addition to bisphosphonates’ plateauing efficacy, their long-term use has been shown to be associated with atypical femoral fractures (AFFs) ^4^. Large doses of intravenous bisphosphonates for the treatment of cancer-associated bone diseases are associated with an increased risk of osteonecrosis of the jaw (ONJ). The incidence of ONJ in subjects whose osteoporosis was treated with low doses of oral or intravenous bisphosphonates is very low, and there is a report suggesting that the incidence of ONJ is not increased among patients whose osteoporosis was treated with bisphosphonate ^5^. Because of these adverse events, a drug holiday is proposed for some patients receiving bisphosphonates ^6^. From those results, it is clear that there are several limitations for nitrogen-containing bisphosphonates, although they certainly increase BMD and prevent fractures, especially during the first several years.

The anti-resorptive activity of bisphosphonates is determined by the balance among several processes, and the behavior of each bisphosphonate is quite different. These include (a) binding of bisphosphonates in the circulation to the mineralized surface of bone matrix, (b) release from the bone surface under an acidic environment by osteoclastic bone resorption, and (c) inhibition of farnesyl pyrophosphate synthase (FPPS) after being taken up by osteoclasts ^7^. FPPS is a rate-limiting enzyme for the formation of geranylgeranyl pyrophosphate, which prenylates and activates small GTP-binding proteins such as Rab, Rho, and Rac, and inhibition of FPPS leads to the suppression of osteoclastic bone resorption. Thus, if the affinity of a bisphosphonate for the mineralized surface of bone under the circulating blood pH is low, only an insufficient amount of bisphosphonate could be accumulated on the bone surface. Alternatively, even though a low amount of bisphosphonate is accumulated on the bone surface, if the affinity of bisphosphonate is low at the Howship’s lacunae under an acidic environment, enough bisphosphonate could be released and taken up by osteoclasts. If a similar amount of bisphosphonate is taken up by osteoclasts, the anti-resorptive activity of bisphosphonate would be determined by the affinity and inhibitory activity of that bisphosphonate for FPPS.

On the mineralized bone surface, hydroxyapatite crystal is bound to collagen matrix, and the affinity of bisphosphonate for the bone surface is determined by its affinity for hydroxyapatite. The affinity for hydroxyapatite at neutral pH is highest for zoledronic acid, followed by alendronate and minodronate, and is lowest for risedronate followed by ibandronate. Under acidic pH, the affinity for hydroxyapatite is highest for alendronate, followed by zoledronic acid and risedronate, and is lowest for minodronate ^8^. Given the long-term accumulation of bisphosphonate in bone, the ideal property of bisphosphonate would be low affinity at neutral pH with less accumulation on the bone surface but low affinity at acidic pH with enough bisphosphonate to be released from bone and strong inhibitory activity to FPPS with inhibition of osteoclastic bone resorption ^9^. Most long-term adverse events such as AFF were reported using alendronate or zoledronic acid, which have high affinity to mineralized bone at neutral and acidic pH, but there are bisphosphonates such as minodronate and risedronate that have physico-chemical characteristics that are substantially different from those of alendronate or zoledronic acid. Therefore, we have to take into consideration that the long-term efficacy on vertebral and non-vertebral fractures, as well as safety issues such as ONJ and AFF, may be different among bisphosphonates with different pharmacological and physico-chemical characteristics.

## Denosumab

Receptor activator of nuclear factor-kappa B ligand (RANKL) expressed in osteoblastic cells binds to its receptor, RANK, on the surface of osteoclasts and their precursor cells. The binding of RANKL to RANK is essential for osteoclast formation, activity, and survival. Denosumab is a human monoclonal antibody against RANKL and has been used for patients with osteoporosis, bone metastases, multiple myeloma, and giant cell tumor of bone. The pivotal phase 3 clinical trial Fracture REduction Evaluation of Denosumab in Osteoporosis every 6 Months (FREEDOM) in postmenopausal women with osteoporosis indicated that denosumab prevented vertebral, non-vertebral, and hip fractures ^10^. There was a close relationship between femoral neck BMD change and fracture risk reduction ^11^. Denosumab showed long-term continuous effects on lumbar and proximal femoral BMD and on vertebral and non-vertebral fracture prevention for up to 10 years ^12, 13^. Denosumab also increased BMD of 1/3 radius consisting of mainly cortical bone almost in a linear fashion. This continuous increase of radial BMD was not reported in patients receiving oral bisphosphonates ^14^. These results demonstrate that denosumab has potent effects on not only trabecular bone but also cortical bone, effects that were not observed using bisphosphonates. Long-term use of denosumab also has anti-fracture effects different from those of bisphosphonates. The yearly incidence of non-vertebral fractures was lower in patients who continued denosumab in the FREEDOM extension study. Whereas the annual incidence of non-vertebral fractures was more than 2% in the first 3 years of denosumab treatment in the FREEDOM study, it became less than 2% during 4 to 7 years of denosumab treatment and 0.7% in year 8 ^13^. These results are consistent with the assumption that the prevention of non-vertebral fractures by denosumab becomes more prominent with long-term use of denosumab. However, it should be noted that a randomized controlled trial was performed for 3 years in the FREEDOM study, and there were no placebo controls in the extension study.

The clearance of denosumab from the blood circulation is very slow because it is not excreted from the kidney or metabolized in the liver but is cleared by the reticuloendothelial system. Consistent with the gradual decrease in the circulating denosumab concentration, bone resorption markers start to increase 4 months after denosumab injection and keep increasing toward the next injection at 6 months ^15^. RANKL expression by osteoblasts and osteocytes is expected to increase at sites of bone with microcracks or deteriorated strength. Thus, there is a possibility that after 4 to 6 months of denosumab injection, bone remodeling can take place, especially where RANKL expression is high. Such a possibility needs to be tested in the future.

Because denosumab rapidly inhibits RANKL-induced bone resorption, if patients do not receive enough vitamin D or calcium or both, there is a risk of hypocalcemia, especially during the first week after administration. Denosumab is also reported to be associated with an increased risk of AFF and ONJ. The non-accumulating, reversible nature of the action of denosumab also causes a rapid increase in bone turnover after discontinuation, resulting in a rapid loss of bone and a possible increase in fractures ^16^. Thus, treatment with other anti-resorptive drugs is required after discontinuation of denosumab. It should also be noted that switching to teriparatide after denosumab treatment further enhances bone remodeling and causes a temporal reduction in vertebral and proximal femoral BMD and a more prolonged reduction in radial BMD ^17^.

## Teriparatide

Teriparatide is a peptide corresponding to the 34 N-terminal amino acids of PTH. Daily subcutaneous injections of teriparatide were shown to increase lumbar BMD and prevent vertebral and non-vertebral fractures ^18^. Teriparatide treatment markedly increases trabecular bone mass but is associated with bone loss at sites composed of mainly cortical bone, such as distal radius. After several months of teriparatide treatment, teriparatide stimulates bone resorption by increasing bone remodeling ^18^. This enhanced bone resorption causes reduced bone mass and increased cortical porosity in peripheral bones ^19^. The use of teriparatide is limited to 2 years in any life span, and this is because of the development of osteosarcoma in pre-clinical animal studies and the decrease of effects to increase BMD. Because long-term teriparatide use is currently not possible, it is necessary to gain maximal benefit of teriparatide during the 2 years. Previous studies indicated that a combination of alendronate with teriparatide does not have an overall superior effect on BMD ^20, 21^. The combination of zoledronic acid and teriparatide induced faster gain in lumbar and hip BMD than the respective drug alone until 26 weeks. After 52 weeks, lumbar BMD in the teriparatide group and hip BMD in the zoledronic acid group were similar to those in the combination group ^22^. In contrast, the recent Denosumab and Teriparatide Administration (DATA) study indicated that the combination of denosumab and teriparatide produced a more prominent effect on BMD than each drug did alone ^23, 24^. The combination of teriparatide and denosumab may have the strongest anti-fracture efficacy among currently available regimens. As mentioned earlier, the DATA-Switch study indicated that teriparatide treatment after denosumab was associated with transient bone loss in lumbar spine and proximal femur and with prolonged BMD decrease in distal radius ^17^. In contrast, teriparatide followed by denosumab continuously increased the BMD of lumbar spine and proximal femur. Thus, it is necessary to consider the timing of teriparatide use, as well as the order of sequential use of teriparatide, in the long-term management of patients with osteoporosis.

## Abaloparatide

Abaloparatide is a synthetic analog of the 34 N-terminal amino acid region of PTH-related protein (PTHrP) with substitution of eight amino acids from amino acid 22 to 31 and Ala ^34^ to Ala-NH _2_. PTH and PTHrP bind to the same PTH1 receptor through their N-terminal portions. PTHR1 can take two conformations, R(0) and RG; R(0) binding results in prolonged signaling, whereas RG binding causes more transient responses. Abaloparatide binds more preferably to the RG conformation and is shown to cause a more transient response than does teriparatide ^25^. The more transient action of abaloparatide via binding to the RG conformation of PTHR1 appears to favor the anabolic effect of abaloparatide with fewer bone resorptive effects. Because abaloparatide is currently under development, most of its clinical data come from a phase 3 clinical study (Abaloparatide Comparator Trial in Vertebral Endpoints [ACTIVE]). In that study, abaloparatide was shown to increase BMD and reduce vertebral and non-vertebral fractures in postmenopausal women with osteoporosis over 18 months ^26^. The reduction of non-vertebral fractures by abaloparatide was significant when compared with placebo, but there was no difference in non-vertebral fractures between the teriparatide and placebo groups. The effects of abaloparatide on the increase in BMD of lumbar spine and proximal femur were shown to be greater than those of teriparatide. Hypercalcemia was lower in the abaloparatide than the teriparatide group ^26^. The superior anti-fracture effects, especially on non-vertebral fractures with less hypercalcemic effect, may render abaloparatide a safe and effective anabolic agent when approved for the treatment of osteoporosis.

## Romosozumab

Canonical Wnt signaling plays a pivotal role in maintaining bone homeostasis. Mouse and human genetic data indicate that canonical Wnt–β-catenin signaling enhances osteoblastic bone formation and inhibits bone resorption via direct and indirect mechanisms. Enhanced Wnt–β-catenin signaling in osteoblastic lineages increases bone mass, whereas reduced signaling decreases bone mass ^27^. Although Wnt signaling ubiquitously affects almost all types of cells, Wnt–β-catenin signaling in bone is controlled by sclerostin, a glycoprotein selectively secreted from osteocytes. Sclerostin is an inhibitor of Wnt signaling and potently inhibits bone formation. Romosozumab is a humanized monoclonal antibody against sclerostin. Romosozumab is also under clinical development, and a phase 3 study demonstrated that romosozumab was shown to markedly increase the BMD of lumbar spine by 13.3% and proximal femur by 6.8% in just 12 months and prevent vertebral and clinical fractures in postmenopausal women with osteoporosis ^28^. Although non-vertebral fractures were not significantly reduced by 12-month romosozumab treatment, the low rate of non-vertebral fracture in the placebo group among the Latin American participants might have affected these results. In a *post hoc* analysis including only subjects outside Latin America, there was a significant 42% reduction in non-vertebral fractures in 1 year because of the higher incidence of non-vertebral fractures in the placebo group (2.7%) ^28^. Among bone turnover markers, a bone formation marker, PINP, increased rapidly 14 days after the first injection and gradually decreased to baseline by 9 months, and there was a transient increase after repeated romosozumab injections ^28^. A bone resorption marker, β-CTX, decreased rapidly on day 14 after the first injection and remained suppressed for 12 months ^28^. Thus, romosozumab increased bone formation and suppressed bone resorption. It must be emphasized that this therapeutic profile is unique to romosozumab. Given the robust anti-fracture effect after only 12 months of treatment, romosozumab can become a strong therapeutic option for the treatment of osteoporosis.

## Conclusions

There are differences in the duration of anti-fracture effect among the currently available anti-resorptive agents. It is important to analyze the difference in the mode of action, efficacy in fracture prevention, and safety profiles of these agents in order to develop safe and effective management of patients with osteoporosis. After the report on ONJ and AFFs among bisphosphonate and denosumab users, there has been increasing concern about the use of drugs for osteoporosis treatment. The current crisis of osteoporosis treatment, owing to the reduction in the number of patients under treatment and the increase in hip fractures, can be overcome by these efforts ^29^. The development of new bone-forming agents such as abaloparatide and romosozumab will increase treatment choice in patients with various severities of osteoporosis and will facilitate the understanding of the necessity and rationale for the combined or sequential treatment of osteoporosis.

## Abbreviations

AFF, atypical femoral fracture; BMD, bone mineral density; DATA, Denosumab and Teriparatide Administration; FPPS, farnesyl pyrophosphate synthase; FREEDOM, Fracture REduction Evaluation of Denosumab in Osteoporosis every 6 Months; ONJ, osteonecrosis of the jaw; PTH, parathyroid hormone; PTHrP, parathyroid hormone-related protein; RANK, receptor activator of nuclear factor-kappa B; RANKL, receptor activator of nuclear factor-kappa B ligand.
